# Computational studies of DNA sequencing with solid-state nanopores: key issues and future prospects

**DOI:** 10.3389/fchem.2014.00005

**Published:** 2014-02-21

**Authors:** Lijun Liang, Qi Wang, Hans Ågren, Yaoquan Tu

**Affiliations:** ^1^Division of Theoretical Chemistry and Biology, School of Biotechnology, KTH Royal Institute of TechnologyStockholm, Sweden; ^2^Department of Chemistry and Soft Matter Research Center, Zhejiang UniversityHangzhou, China

**Keywords:** solid-state nanopore, graphene nanopore, computer modeling, bio-nanotechnology, DNA sequencing, theory, molecular simulation

## Abstract

Owing to the potential use for real personalized genome sequencing, DNA sequencing with solid-state nanopores has been investigated intensively in recent time. However, the area still confronts problems and challenges. In this work, we present a brief overview of computational studies of key issues in DNA sequencing with solid-state nanopores by addressing the progress made in the last few years. We also highlight future challenges and prospects for DNA sequencing using this technology.

## Introduction

Sequencing the human genome allows us to better understand the relationships among diseases, inheritance, and individuality. Although the increasing need for cheap and fast genome sequencing has promoted the development of new sequencing technologies, the cost of genomic sequencing is still far from the ideal price point of “the $1000 genome” (Porcu et al., 2013). This means that even cheaper and faster sequencing methods need to be developed. For this purpose, “single molecule” sequencing has been considered to be a promising technology (Harding and Keller, 1992; Pushkarev et al., 2009). In particular, “single molecule” sequencing with nanopores has been recommended as the next generation platform for DNA sequencing (Rhee and Burns, 2006; Maitra et al., 2012; Ku and Roukos, 2013).

**Figure 1:**
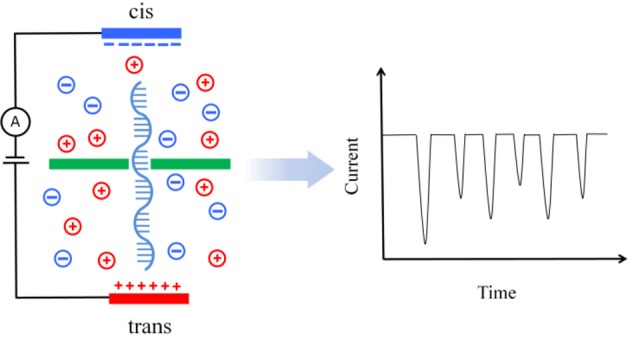
**Illustration of translocation of a DNA molecule through a nanopore device**. The pore divides the solution into two parts. A bias voltage is applied across the pore to generate an electric field perpendicular to the wall and to drive the DNA through the pore. The DNA translocation is monitored by the blockade current (schematically illustrated on the right) associated with the nucleotides through the nanopore.

The basic principle of DNA sequencing with a nanopore is illustrated in Figure 1. A DNA molecule, either double strand (dsDNA) or single-strand (ssDNA), dispersed in a salt solution (such as a KCl solution) is driven by an applied electric field to pass through a nanopore for sequencing. As the DNA translocates through the nanopore, the flow of ions is interrupted and the ionic current is blocked as a function of time. This phenomenon was first observed by Kasianowicz et al. in a study of a DNA molecule translocation through the α-haemolysin membrane pores (Kasianowicz et al., 1996). Later, it was found that the ionic current was associated with the particular nucleotides [adenine (A), cytosine (C), guanine (G), and thymine (T)] passing through the nanopore (Clarke et al., 2009). This makes it possible for nucleotides to be distinguished in terms of the detected current. DNA sequencing with a nanopore is thus to identify the composition of a DNA molecule from the pattern of the ionic current as the DNA passes through the nanopore in a salt solution (Purnell and Schmidt, 2009).

Both biological and solid-state nanopores can be used for DNA sequencing. It has been reported that sequence information can be obtained with biological nanopores (Cherf et al., 2012; Manrao et al., 2012). This reflects that DNA sequencing with biological nanopores has achieved a significant progress. However, the fact that the lipid membrane used to fix biological nanopores is delicately sensitive to the temperature, *pH* value and salt concentration makes it difficult to control their stability. For this reason, solid-state nanopores have become the promising alternative to biological nanopores (Merchant et al., 2010; Schneider et al., 2010; Garaj et al., 2013).

DNA sequencing with solid-state nanopores has experienced fast development in recent years (see for example, Venkatesan and Bashir, 2011a,b; Yang et al., 2013 for reviews). Yet, it still confronts problems, such as that the error rate is too large (Schadt et al., 2010) and the DNA translocation speed is too high. Theoretical studies can help us to understand the molecular details of DNA translocation through a nanopore. In this mini-review, we focus on the recent progress in computational studies on key issues in DNA sequencing with solid-state nanopores, such as control of the DNA translocation speed and the electronic signature of the identity of bases. We also address future challenges and prospects of DNA sequencing with this technology.

## Control of the DNA translocation speed in solid-state nanopores

One of the main challenges in DNA sequencing with a solid-state nanopore is to reduce the speed of the DNA molecule passing through the nanopore (Kowalczyk and Dekker, 2012). The DNA translocation speed is still too high using the currently available solid-state nanopores. However, the development in the field indicates that ideal speeds are expected to be achieved around 2015 (Venkatesan and Bashir, 2011a). DNA translocation through a nanopore is affected by many factors, such as the solvent viscosity, ion concentration, surface charge, and diameter of the pore. In theory, assuming that the end effects of a nanopore are negligible and that a DNA molecule can only move along the center axis of the pore, we can determine the translocation speed of the DNA in an applied electric field by solving a set of coupled differential equations (Ghosal, 2007; Kejian et al., 2009), i.e., the Stokes equation for the flow of the DNA to the pore surface and the Poission equation for the distribution of the electric potential in the pore. The resulting translocation speed is simply given as (Luan et al., 2012a):

(1) $$\nu =\varepsilon \times \frac{\zeta_{d}-\zeta_{w}}{\eta }E,$$

where ε is the dielectric constant of the electrolyte, ζ_*d*_ and ζ_*w*_ are the zeta potentials on the DNA and pore surfaces, respectively, η is the viscosity of the electrolyte, and *E* is the bias electric field. According to Equation (1), we can tune the electric field, solvent viscosity, etc. to control the speed. Experiments as well as theoretical calculations have been carried out to study the translocation time by changing the electric field or solvent viscosity (Japrung et al., 2010; Luan et al., 2012a).

By lowering the temperature of a graphene nanopore, Fologea et al. decreased the translocation speed by a factor of ~2 (Fologea et al., 2005). Luan et al. demonstrated that the translocation speed of an ssDNA molecule could be reduced by 10 times in a S_i_O_2_ nanopore in a glycerol solvent (Luan et al., 2012a,b). By decreasing the size of the counter ions, for example from K^+^ to Na^+^ to Li^+^, Dekker et al. revealed that the translocation time of a DNA molecule through a solid-state nanopore increased significantly (Kowalczyk et al., 2012). Molecular dynamics (MD) simulations indicated that Li^+^ binds to DNA more strongly than K^+^ does. The stronger interaction of the ions with the DNA thus creates a much stronger drag because the movements of the ions and the DNA occur in the opposite directions (Kowalczyk et al., 2012).

In the above mentioned studies, the DNA translocation speed in the solid-state nanopores was indeed decreased. However, the variations in the translocation dynamics due to the DNA-pore interactions (Aksimentiev et al., 2004; Branton et al., 2008; Gierhart et al., 2008) were not reduced and accordingly the identification of different bases could not be enhanced. Computer modeling methods have been used to study the change of translocation dynamics of DNA passing through nanopores. Employing steered molecular dynamics (SMD) simulations, Luan et al. found that the translocation time was increased by chemical modification of the nanopore surface (Luan et al., 2010). The work of Luan et al. has enhanced our understanding of factors in decreasing the translocation speed in solid-state nanopores. By using a continuum-based model, Zhang et al. indicated that the local permittivity environment could reduce the DNA translocation speed (Zhang et al., 2012). Theoretical modeling has also been used to study the translocation time. Using the Lubensky-Nelson model (Lubensky and Nelson, 1999), Reimann et al. showed that the time distribution derived from the model matches the experimental data very well (Reimann et al., 2012). From Monte Carlo simulations, Polson et al. found that the translocation time is proportional to (*N* − *N_P_*)^2^, i.e., 〈τ〉 ∝ (*N* − *N_P_*)^2^ for a sufficiently narrow pore, where *N* means the polymer length and *N_p_* is the average number of monomers in the nanopore (Polson and Mccaffrey, 2013). In addition, a theoretical study reported by He et al. indicated that by utilizing the cross-pore thermal gradient, the DNA translocation speeds could be orders of magnitude slower than the electrophoretic counterpart (He et al., 2012).

## Base identification with solid-state nanopores

There have been many theoretical studies on detecting nucleotide differences by solid-state nanopores (Postma, 2010; Wells et al., 2012). As indicated from computer simulations, A-T and G-C base pairs could be discriminated using graphene nanopores (Sathe et al., 2011). Through theoretical modeling, Liang et al. illustrated that the DNA translocation time could be extended and the discrimination of the bases could be improved by narrowing the nanopores (Liang et al., 2013). Aksimentiev et al. showed that the hydrophobic interactions of the nucleotides with the graphene membrane led to a dramatic reduction in the conformational fluctuations of the nucleotides in the pore and that the resulting ionic current blockades were different for different DNA nucleotides (Wells et al., 2012). The results from SMD simulations indicated that each nucleotide, except for cytosine and thymine, in an ssDNA could be identified and characterized by the sub-2-nanometer nanopore (Qiu and Guo, 2012). Garaj et al. pointed out that a molecule-hugging graphene nanopore could have a resolution higher than 0.6 nm along the length of the molecule (Garaj et al., 2013). In addition, theoretical calculations have also shown that different nucleotides can be discriminated by the difference in the conductance spectra (Nelson et al., 2010; Avdoshenko et al., 2013). In these studies, the read lengths of the DNA molecules were very short (shorter than 100 base pairs) and the data may therefore not have reflected the complexity in experiment where the number of base pairs is often more than 1000. However, theoretical studies significantly enhance our understanding of base identification for DNA sequencing with solid-state nanopores, especially with graphene nanopores, and can lay a solid theoretical foundation for DNA sequencing with nanopores in the future.

## Future challenges and outlook

In using solid-state nanopore-based devices for DNA sequencing, the major concerns are the range of DNA lengths, the sequencing speed, and the error rate in the sequencing. To decrease the DNA translocation speed we can adopt some measures such as using more sticky solvents or decreasing the temperature. However, by these measures, the DNA translocation dynamics, which originates from the DNA-pore interactions, cannot be changed. The most promising approach to change the DNA translocation dynamics is to change the nanopore material because we can evidently not change the properties of the DNA. A few studied ways to deal with this issue include those changing the pore diameter (Liang et al., 2013), pore geometry (Aksimentiev et al., 2004; Venkatesan et al., 2009), the charge on the pore surface (Luan and Aksimentiev, 2008), and using surfaces modified by SAM (Luan et al., 2010).

Normally, solid-state nanopores are made of silicon nitride, aluminum oxide, or silicon oxide. In recent years, graphene nanopores have been suggested as a potential material for sequencing DNA because of their unique electric and mechanical properties. However, our understanding of DNA sequencing with graphene nanopores is still rather limited, necessitating further studies in this area.

Simulations can greatly enhance our understanding of the atomic details of DNA translocation dynamics and DNA-pore interactions. We have addressed the use of simulations to study the DNA translocation in solid-state nanopores. Many simulation studies have captured important physical properties specific to DNA sequencing with nanopores. However, the underlying simulation models are relatively simple, and may not reflect the complexity of the real systems. Compared to the experimental conditions used, the applied voltages in the simulations are often too large, leading to too high translocation speeds. This may produce unpredictable influence on the translocation dynamics. In a real system, the charge on the nanopore surface can also change with the *pH* value, solvent, and ionic concentration. Furthermore, the nanopore geometry can greatly modulate the drag forces, something that is not considered in most simulations. In addition, the error rate of DNA sequencing has not been assessed in most computational studies since the read lengths of the DNA molecules used in these studies have been very short. All these issues need to be attended in the future. Given so many new opportunities and given the considerable progress we have witnessed in this field, it can be foreseen that studies of DNA sequencing with solid-state nanopores will remain active for years to come.

### Conflict of interest statement

The authors declare that the research was conducted in the absence of any commercial or financial relationships that could be construed as a potential conflict of interest.
